# The chemokine, macrophage inflammatory protein-2γ, reduces the expression of glutamate transporter-1 on astrocytes and increases neuronal sensitivity to glutamate excitotoxicity

**DOI:** 10.1186/1742-2094-9-267

**Published:** 2012-12-12

**Authors:** Jie Fang, Deping Han, Jinsheng Hong, Qi Tan, Yeping Tian

**Affiliations:** 1 Department of Dermatology, Yangpu District Central Hospital, Shanghai, 200090, China; 2 First Affiliated Hospital, Fujian Medical University, Fuzhou, Fujian, 350005, China; 3 Institute of Immunology and National Key Laboratory of Medical Immunology, Second Military Medical University, Shanghai, 200433, China

**Keywords:** Glutamate transporter, Chemokines, MIP-2γ, Astrocyte, Lipid rafts

## Abstract

**Background:**

Changes in glutamatergic neurotransmission via decreased glutamate transporter (GLT) activity or expression contributes to multiple neurological disorders. Chemokines and their receptors are involved in neurological diseases but the role of chemokines in the expression of glutamate transporters is unclear.

**Methods:**

Primary astrocytes were prepared from neonatal (<24 hours old) SJL/J mouse brains and incubated with 5 μg/ml lipopolysaccharide (LPS) or 50 ng/ml tumor necrosis factor α (TNF-α) for 24 hours. Soluble macrophage inflammatory protein-2γ (MIP-2γ) in culture supernatants was determined using a sandwich ELISA. The MIP-2γ effect on the expression of GLT-1 was measured by quantitative RT-PCR, flow cytometric analysis or western blot assay. Detergent-resistant membranes from astrocytes were isolated on the basis of their ability to float in density gradients. Raft-containing fractions were tracked by the enrichment of caveolin-1 and the dendritic lipid raft marker, flotillin-1. Cell viability was determined by measuring either the leakage of lactate dehydrogenase or the reduction of 3-(4,5-dimethylthiazol-2-yl)- 2,5-diphenyltetrazolium bromide by viable cells and confirmed by visual inspection.

**Results:**

The production of the chemokine MIP-2γ by mouse cortical astrocytes increased significantly after stimulation with LPS or TNF-α *in vitro*. Astrocytes over-expressing MIP-2γ down-regulated the expression of GLT-1 at the mRNA and protein level and caused redistribution of GLT-1 out of the lipid rafts that mediate glutamate uptake. We used pharmacological inhibitors to identify the downstream signaling pathways underlying MIP-2γ activity. We also found complementary results by knocking down MIP-2γ activity in astrocytes with MIP-2γ small interfering RNA (siRNA). MIP-2γ overexpression in astrocytes enhanced the neuronal toxicity of glutamate by decreasing GLT-1 activity, but MIP-2γ itself was not toxic to neurons.

**Conclusions:**

These results suggest that MIP-2γ mediates the pathogenesis of central nervous system disorders associated with neutrophil infiltration in the brain and decreased GLT-1 activity.

## Background

Glutamate is the major excitatory neurotransmitter in the mammalian central nervous system (CNS). At high extracellular levels, this excitatory amino acid is an extremely potent neurotoxin. Extracellular glutamate concentrations in the CNS are regulated by a family of high-affinity, Na^+^-dependent glutamate transporters. Five subtypes of transporters, EAAT1–EAAT5 (excitatory amino acids transporters 1–5), have been identified, two of which, the glutamate transporter GLT-1 (also known as EAAT-2) and glutamate-aspartate transporter GLAST (also known as EAAT-1 or GLT-2), are predominantly present on astrocytes and are the major glutamate transporters in the CNS [1]. Malfunction or aberrant expression of these glutamate transporters can cause accumulation of toxic concentrations of glutamate and trigger neurodegeneration. Reduced GLT-1 protein expression occurs in brain injury or ischemia, Alzheimer’s disease, Huntington’s disease, HIV-1-associated dementia (HAD), and experimental autoimmune encephalomyelitis (EAE) [2-5]. In EAE, for example, GLT-1 and GLAST proteins are down-regulated in the spinal cord at the peak of disease symptoms, with no recovery in expression after remission. These data emphasize the importance of the astrocyte GLT-1 transporter for normal brain function and its contribution to multiple CNS pathologies.

Astrocytes are often associated with the pathogenesis of infectious and immune inflammatory responses involving the CNS and are a major source of chemokines, such as monocyte chemoattractant protein-1 (MCP-1), macrophage inflammatory protein-1α (MIP-1α), inflammatory protein-10 (IP-10), RANTES and MIP-2 [6,7]. Proinflammatory mediators such as lipopolysaccharide (LPS), tumor necrosis factor α (TNF-α), and IL-1 induce astrocytes to release many of these chemokines *in vitro*[8,9]. Chemokines and their receptors are involved in neurological diseases, including multiple sclerosis, Alzheimer’s disease, HAD, and cerebral ischemia [10-12]. However, the role of chemokines in the expression of glial glutamate transporters is unclear.

A novel CXC chemokine, MIP-2γ (also known as CXCL14) was identified and characterized from a human dendritic cell (DC) cDNA library [13]. MIP-2γ mRNA is widely and constitutively expressed in normal tissues, including the brain. MIP-2γ exhibited potent chemotaxis on neutrophils, was less active on DCs, and inactive on monocytes, NK cells, and T and B lymphocytes [13]. We found that MIP-2γ mRNA expression within the CNS of EAE mice varied at the onset, peak, remission, and relapse [14]. But the origin and role of MIP-2γ in inflammatory disorders of the brain are still unclear.

Therefore, we analyzed the effect of MIP-2γ on GLT-1 and GLAST expression in astrocytes, as well as their redistribution into functional raft microdomains. We also measured changes in glutamate uptake in response to MIP-2γ overexpression and dissected the MIP-2γ signaling pathways. Finally, we evaluated whether MIP-2γ overexpression enhanced neuronal sensitivity to glutamate toxicity.

## Materials and methods

### Expression vectors construction

The mouse wild-type MIP-2γ cDNA [13] was subcloned into pAAV-IRES-hrGFP (Stratagene, La Jolla, CA, USA) to create the MIP-2γ expression vector, pAAV-MIP-2γ-hrGFP. MIP-2γ cDNA sequences were confirmed by DNA sequencing. To construct an RNAi vector to silence the MIP-2γ gene, we designed and synthesized three double-stranded oligonucleotides targeting three different sites of the MIP-2γ cDNA (position 440–460, 465–485 and 574–594, AF252873) that could generate hairpin small interfering RNAs (siRNA-1, siRNA-2 and siRNA-3, respectively). The selection of the coding sequences for siRNA was analyzed by BLAST to ensure that they did not have significant sequence homology with other genes. Then, the oligonucleotides were inserted into pBS/U6 (gift of Yang Shi, Harvard Medical School, Boston, MA, USA) according to the method of Sui *et al*. [15]. After confirmation by sequencing, RNAi vectors were transfected into astrocytes with Polyfect (QIAGEN, Valencia, CA, USA) according to the manufacturer’s instructions. The negative control plasmid (pBS/U6-con) contains a scrambled sequence (GCTGTCTGATCAATGGACGAC) that does not show significant homology to mouse gene sequences.

### Cell culture and treatment

Primary astrocytes were prepared from neonatal (<24-hours old) SJL/J mouse brains using methods similar to those described previously [16,17]. Primary glial cell cultures were maintained in (D)MEM (Gibco, Carlsbad, CA, USA) supplemented with 10% FCS, 6 mg/ml glucose, and 5 μg/ml bovine pancreas insulin (Sigma-Aldrich, St. Louis, MO, USA), referred to as complete medium, in 10% CO_2_ at 37°C. After 11 days, the flasks were agitated on an orbital shaker for 14 hours at 250 rpm at 37°C, and the nonadherent oligodendrocyte and microglial cells were removed. Cortical astrocytes were purified from the primary mixed glial cell culture by three to four repetitions of trypsinization and replating. The purity of astrocytes was more than 95% when determined by indirect immunofluorescence using an anti-glial fibrillary acid protein (GFAP) antibody. To increase surface expression of glutamate transporters, astrocyte cultures were treated with 250 μM dibutyryl cAMP (dBcAMP) (Sigma) for 7 days before experimentation.

Neuron cultures were prepared from SJL/J mice at embryonic day 17. In brief, the cortices were dissected and freed of meninges. Cortical fragments were incubated with 0.25% trypsin and 20 μg/ml DNase I in PBS at 37°C for 15 minutes. The cortical fragments were then dissociated into single cells by pipetting, and the cells were suspended in Neurobasal-A medium containing a B27 serum-free supplement (Invitrogen, Grand Island, NY, USA) and plated onto poly-D-lysine (50 μg/ml)-coated plates. Twenty-four hours later, the cultures were treated with 5 μM cytosine arabinoside (Sigma) *in vitro* for 72 hours to prevent proliferation of other cell types. More than 95% of the cells were positive for staining with the neuron-specific microtubule-associated protein 2 (MAP-2) antibody (Sigma).

Induction with LPS and TNF-α was performed on 1×10^6^/ml astrocytes cultured for 24 hours with or without 5 μg/ml LPS or 50 ng/ml TNF-α, with the supernatants collected for an MIP-2γ ELISA assay and the remaining cells washed three times with PBS and collected for RNA or protein extraction. Astrocytes (1×10^6^/ml) were transfected with the plasmid pAAV-IRES-hrGFP or the plasmid pAAV-MIP-2γ-hrGFP alone or combined with either the pBS/U6 vector or the plasmid MIP-2γ siRNA using Polyfect (QIAGEN) according to the manufacturer’s instructions. Media were collected three days after transfection and the remaining cells were washed three times with PBS for RNA or protein extraction. The plasmid pAAV-IRES-hrGFP containing the hrGFP gene was used as a control for transient transfection. The expression of hrGFP by astrocytes was analyzed by fluorescence microscopy (Leica, Deerfield, IL, USA) using a ×20 objective, and the data were acquired with a Sony digital charge-coupled device camera and processed by Adobe PHOTOSHOP software.

For neuron/astrocyte (N/A) co-culture experiments, neurons were plated as normal in 24-well trays and maintained in culture for 11 days before addition of astrocytes. Transfected or untransfected astrocytes were plated in tissue culture inserts (BD Falcon, Franklin Lakes, NJ, USA) at 10^4^ cells/insert with 3.0 μm pores. Before assaying neuron survival, the inserts were removed and 3-(4,5-dimethylthiazol-2-yl)- 2,5-diphenyltetrazolium bromide (MTT; Sigma) assays or lactate dehydrogenase (LDH) release tests were performed on the neurons. Immunostaining against the cytoskeleton constituent, MAP-2, confirmed their neuron identity.

### MIP-2γ ELISA

Secreted, soluble MIP-2γ in culture supernatants was determined using a sandwich ELISA with a monoclonal anti-mouse MIP-2γ antibody [13]. The captured antibody was used at 1 μg/ml and the biotinylated detection antibody was used at 400 ng/ml (R&D Systems Inc. Minneapolis, MN, USA).

### Detergent-free isolation of lipid rafts

Detergent-resistant membranes from astrocytes were isolated on the basis of their insolubility in Triton X-100 at 4°C and their ability to float in density gradients as described previously [18,19]. Astrocytes were homogenized in a homogenizer in 1.5 ml of PTN 50 buffer (50 mM sodium phosphate, pH 7.4, 1% Triton X-100, 50 mM NaCl) containing 10 mM dithiotheritol, 1 mM phenylmethylsulfonyl fluoride, 5 μg/ml leupeptin, and 1 μg/ml pepstatin A, and centrifuged at 12,000 rpm for 3 minutes. The supernatant was placed at the bottom of an ultracentrifugation tube and mixed with an equal volume of 80% (w/v) sucrose. The samples were overlaid with 30% (w/v) and 5% (w/v) sucrose, respectively. The gradient was centrifuged at 55,000 rpm for 2 hours at 4°C with a TLS 55 Beckmann swing rotor. Ten fractions were collected from the top of the gradient and protein levels quantified. An equal volume of each fraction (30 μl) was diluted in a loading buffer and used for immunoblotting. Fractions corresponding to raft microdomains (fraction numbers 3, 4 and 5) and fractions corresponding to detergent-soluble material (fraction numbers 8, 9 and 10) were pooled before loading on the same acrylamide gel. Six wells were pooled for each condition, and only the fractions corresponding to raft and soluble proteins were studied. Raft-containing fractions were tracked by the enrichment of the cholesterol binding protein, caveolin-1, and the dendritic lipid raft marker, flotillin-1.

### RT-PCR and real-time quantitative PCR analysis

The primer sets used in RT-PCR for MIP-2γ, GLT-1, GLAST, GFAP, and β-actin were designed with Oligo software (Table 1). Total RNA was isolated from astrocytes using Trizol reagent (Life Technologies, Carlsbad, CA, USA). A total of 20 μg of RNA was reverse-transcribed by using 200 U per μl of Moloney murine leukemia virus (Promega, Madison, WI, USA) and 2 μg of random hexamer primers (Interactiva, Ulm, Germany). Obtained templates were amplified in a final volume of 50 μl. Cycling conditions comprised an initial denaturation of 3 minutes at 94°C followed by 30 cycles of amplification (at 94°C for 40 seconds, 50°C for 45 seconds and 72°C for 1 minute) and final elongation step at 72°C for 10 minutes in the presence of 20 pmol of primers. Reaction products were separated and visualized with ethidium bromide on a 1.5% agarose gel. Real-time PCR was performed in the Applied Biosystems 7500 Real-Time PCR System software using SYBR GREEN PCR MasterMix (Qiagen). PCR was performed under the following conditions: initial denaturation at 95°C for 15 minutes and 37 cycles of 95°C for 30 seconds, 60°C for 30 seconds, and 72°C for 20 seconds. The generation of specific PCR products was confirmed by melting curve analysis. Each reaction was run in triplicate. The expression of GLT-1 was normalized against β-actin by the comparative threshold cycle (ct) method using the following formula: fold difference in expression = 2^–(Δct of target gene–Δct of reference)^. Primer sequences for real time PCR were: GLT-1, 5^′^-GGCAATCCCAAACTCAAGAAGC-3^′^ (forward) and 5^′^-GTCACTGTCTGAATCTGCTGGAAAC-3^′^ (reverse); β-actin, 5^′^-GCCAACACAGTGCTGTCT-3^′^ (forward), and 5^′^-AGGAGCAATGATCTTGATCTT-3^′^ (reverse).

**Table 1:** **RT**-**PCR primers and predicted product length**

**mRNA**	**Primer sequence**	**Primer length (bp)**	**Product length (bp)**
MIP-2γ	Forward 5'-GCGCGTTGGACGGGTCCAAGT-3'	21	200
	Reverse 5'-GCGAGCACCAAACGCTTCATC-3'	21	
GLT-1	Forward 5'-TTCCAGTCTCATCACAGGGCT-3'	21	463
	Reverse 5'-GCCGAAAGCAATAAAGAATCC-3'	21	
GLAST	Forward 5'-TAAGTATCACAGCCACAGCCG-3'	21	454
	Reverse 5'-GAGTAGGGAGGAAAGAGGAG-3'	20	
GFAP	Forward5'-AGTTACCAGGAGGCACTTGCT-3'	21	484
	Reverse 5'- TCCTGTTCTATACGCAGCCAG-3'	21	
β-actin	Forward 5'-ATCCGTAAAGACCTCTATGC-3'	20	287
	Reverse 5'-AACGCAGCTCAGTAACAGTC-3'	20	

### Western blotting

Whole cells were homogenized in radioimmunoprecipitation assay buffer (PBS, 0.1% sodium dodecyl sulfate (SDS), 1% NP-40, 0.5% sodium deoxycholate) and protein concentrations of the samples determined by the bicinchoninic acid method (Pierce) using BSA as a standard. Equal amounts of total protein (5 to 8 μg) were adjusted to similar volumes with loading buffer (10% SDS, 20% glycerin, 125 mM Tris, 1 mM ethylenediaminetetraacetic acid, 0.002% bromphenol blue, 10% β-mercapto-ethanol), denatured by heating at 95°C for 5 minutes, subjected to 10% SDS-PAGE, and then electroblotted onto a nitrocellulose membrane (Amersham) using a minigel and mini transblot apparatus (Bio-Rad, Hercules, CA, USA). The membranes were blocked with 5% nonfat dry milk in TBST buffer (25 mM Tris–HCl pH 8.0, 125 mM NaCl, and 0.1% Tween 20) for 1 hour at room temperature. The blots were then incubated with either anti-MIP-2γ (1:500; R&D Systems), anti-GLT-1 (1:1000; Santa Cruz Biotechnology, Inc., Santa Cruz, CA, USA), anti-GLAST (1:1000; Santa Cruz Biotechnology), anti-GFAP (1:500; Dako, Glostrup, Denmark), anti-flotilin-1 (1:500; Transduction Laboratories, San Diego, CA, USA), anti-caveolin-1 (1:500; Transduction Laboratories), or anti-β-actin (1:1000; Santa Cruz Biotechnology) antibodies diluted in TBS/Tween overnight at 4°C. The blots were incubated with the appropriate horseradish peroxidase-conjugated secondary antibodies (Jackson Immuno-Research, West Grove, PA, USA) for 1.5 hours at room temperature. Membrane-bound secondary antibodies were detected using the chemiluminescence Super Signal procedure (Pierce) according to the manufacturer’s instructions.

### Flow cytometric analysis of GLAST and GLT-1

Cells (1 × 10^7^ cells/ml) were washed twice with PBS, preincubated in PBS/1% BSA (Sigma) for 1 hour at 4°C, and then incubated with unconjugated rabbit polyclonal anti-GLAST and anti-GLT-1 antibodies for 1 hour at 4°C. Subsequently, cells were washed once with PBS/0.1%BSA and stained with fluorescein isothiocyanate-conjugated goat anti-rabbit immunoglobulin G (IgG) (Molecular Probes, Eugene, OR, USA) for 30 minutes. The fluorescence intensity of cells was measured using FACScan (Becton Dickinson, Mountain View, CA, USA).

### [^3^H]-glutamate uptake assay

L-[^3^H] glutamate uptake was determined by the method described by Lee and coworkers (19). In brief, after transfection and supernatant collection, the medium was replaced by 0.5 ml of fresh medium containing 30 μM unlabeled glutamate and 0.025 μCi/ml L-[^3^H]-glutamate (10 nM, 45.0 Ci/mmol; Amersham Biosciences, Buckinghamshire, UK). Uptake was terminated 10 minutes later by removing the supernatant and washing the cells three times with 2 ml of ice-cold PBS containing 5 mM glutamate. Astrocytes were then lysed by 0.5 ml of 1 N NaOH containing 0.1% Triton X-100, and a 300-μl portion was assayed for ^3^H by liquid scintillation counting. The protein content was measured by Lowry’s protein assay using BSA standards in 100 μl of the remaining lysate. Uptake was expressed as cpm per minigram of cellular proteins.

### Cell viability assay

Neurons were co-cultured with untransfected or transfected astrocytes for 4 days; after this time, glutamate (50 μM) was added. After 4 hours, the inserts containing the astrocytes were discarded and neuronal survival was determined by measuring either the leakage of LDH from dead or dying cells into the culture medium or the reduction of MTT by viable cells and confirmed by visual inspection.

Astrocyte conditioned media (AM) derived from different groups was collected and used without freezing by direct application on neuronal cultures. Primary cortical neuronal cultures were seeded onto 96-well plates at 5,000 cells per well in B27-supplemented medium in the presence or absence of different AM (20% to 80% v/v) for 4 days. Medium was then replaced with fresh B27-supplemented medium containing 50 μM glutamate and 4 hours later the cell viability was determined. LDH activity was evaluated by using a commercially available kit (Roche Molecular Biochemicals, Mannheim, Germany) according to the manufacturer’s directions. In parallel experiments, cell viability was assessed by an immunocytochemistry analysis using a MAP-2 antibody to detect neurons.

### Immunocytochemistry

Immunocytochemistry was performed as previously described [20]. Cells plated on sterile glass coverslips coated with poly-d-lysine (50 μg/mL) were fixed with 4% paraformaldehyde in PBS. After incubation with primary antibodies (anti-GFAP 1:100 and anti-MAP-2 1:100; Sigma) overnight at 4°C, coverslips were washed with blocking buffer and incubated with anti-mouse IgG-fluorescein conjugates and then subjected to immunofluorescent microscope analysis. The number of immunolabeled cells was counted manually on fluorescence microscopic images by three independent individuals. All cell countings were location-matched between each well. One frame for the visual field of 100 × 100 μm was used for cell counting. Three different visual fields were chosen randomly. Average values of cell counts were calculated from the pooled data.

## Results

### Inflammatory stimuli increase the expression of MIP-2γ in cultured mouse cortical astrocytes

**Figure 1:**
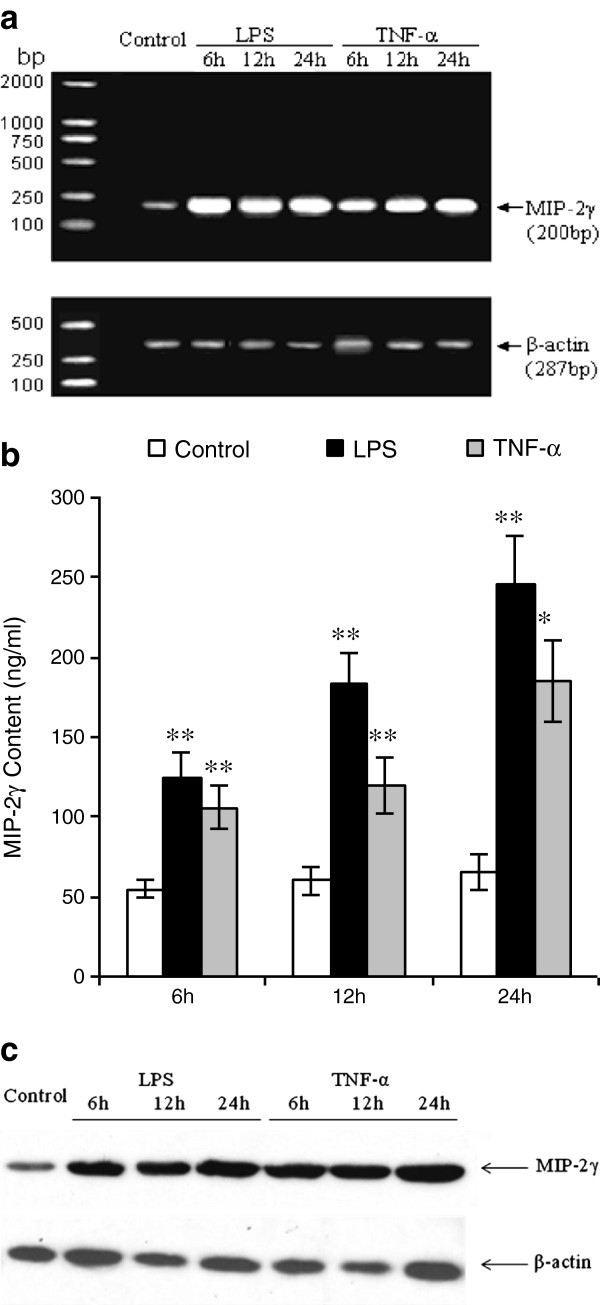
**Relative MIP-2γ mRNA and protein expression in astrocytes.** (**a**) RT-PCR analysis of MIP-2γ expression in purified primary astrocyte cultures. Levels of MIP-2γ mRNA were increased in primary cultures of astrocytes exposed to LPS or TNF-α compared with buffer-treated astrocytes (control). β-actin was used as a positive control. (**b**) MIP-2γ is up-regulated after exposure to LPS or TNF-α, shown by an ELISA for MIP-2γ in the conditioned media of astrocytes. Data are expressed as the mean ± SEM from three to four independent experiments. *P <0.05; **P <0.01 significantly different from buffer-treated control (ANOVA followed by a Dunnett’s test). (**c**) Representative western blot probed for MIP-2γ and β-actin simultaneously. Levels of MIP-2γ in cell lysates derived from primary astrocytes exposed to LPS or TNF-α were increased compared with buffer-treated astrocytes (control). ANOVA, analysis of variance, LPS, lipopolysaccharide; MIP-2γ, macrophage inflammatory protein-2γ; SEM, standard error of the mean; TNF-α, tumor necrosis factor α.

We examined the ability of astrocytes to produce and respond to inflammatory stimuli *in vitro*. Total RNA was extracted from cortical astrocytes that were exposed to LPS (5 μg/ml) or TNF-α (50 ng/ml) for 6, 12, or 24 hours, and subjected to cDNA synthesis and RT-PCR analysis using primers for MIP-2γ and β-actin (Table 1). RT-PCR analysis revealed that buffer-treated astrocytes expressed messages for the housekeeping gene, β-actin, and traces of MIP-2γ, whereas LPS and TNF-α treatment increased MIP-2γ expression (Figure 1a, 1b). Immunoblot analysis also showed that LPS and TNF-α increased MIP-2γ protein expression in astrocytes (Figure 1c).

### Specific knockdown MIP-2γ expression in cultured astrocytes by siRNA

**Figure 2:**
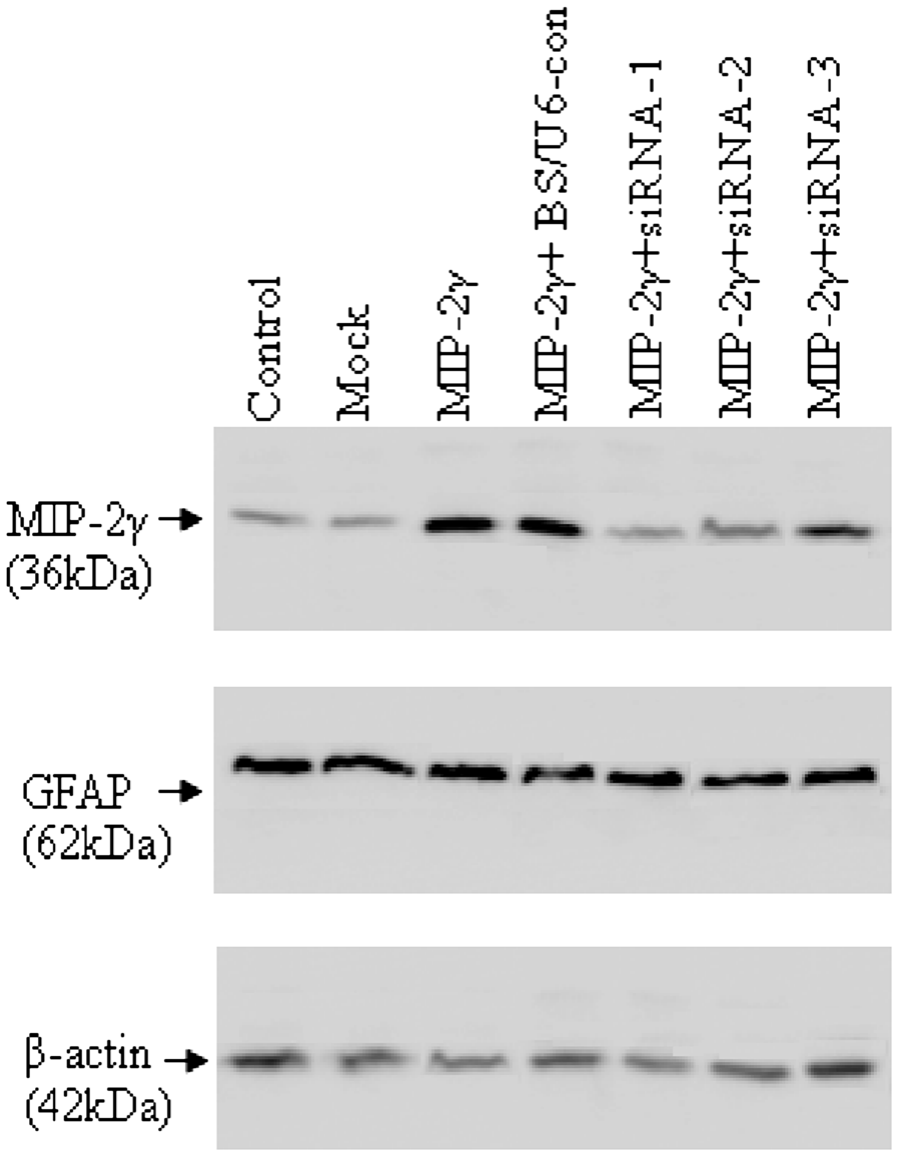
**Specific knockdown of MIP-2γ expression in cultured astrocytes by siRNA.** Representative western blots of MIP-2γ and GFAP. The expression of MIP-2γ was significantly increased after pAAV-MIP-2γ-hrGFP transfection (MIP-2γ) compared with untreated cultures (control) or transfected with pAAV-IRES-hrGFP (mock) vector. This increase in MIP-2γ was inhibited by co-transfection with MIP-2γ siRNAs (MIP-2γ + siRNA-1, MIP-2γ + siRNA-2, and MIP-2γ + siRNA-3), but not by pBS/U6-con (MIP-2γ + BS/U6-con) vector. No significant changes in GFAP occurred. Equal loading was confirmed by exposing the membranes to an anti β-actin antibody. One of four experiments that generated the same result is shown. GFAP, glial fibrillary acid protein; MIP-2γ, macrophage inflammatory protein-2γ; siRNA, small interfering RNA.

We first examined the efficiency of astrocyte transduction using a pAAV-MIP-2γ-hrGFP vector, and at least 80% produced robust green fluorescence within two to three days that did not diminish (for at least two weeks, data not shown, replicated in at least three independent experiments). We then used a siRNA to block MIP-2γ expression. Astrocytes were cultured on 6-well plates and transfected with either MIP-2γ siRNAs or the pBS/U6-con vector together with pAAV-MIP-2γ-hrGFP 24 hours after plating. At 48 hours post-transfection, cell extracts were assayed by immunoblotting for MIP-2γ protein. Transfection with siRNAs directed at specific MIP-2γ mRNA sequences suppressed MIP-2γ protein, especially MIP-2γ siRNA-1 (Figure 2), whereas transfection with the pBS/U6-con did not. In addition, transfection with MIP-2γ siRNAs did not dramatically change levels of GFAP, a marker for astrocyte activation, suggesting that astrocyte function was not disrupted.

### MIP-2γ decreases astrocyte expression of GLT-1

**Figure 3:**
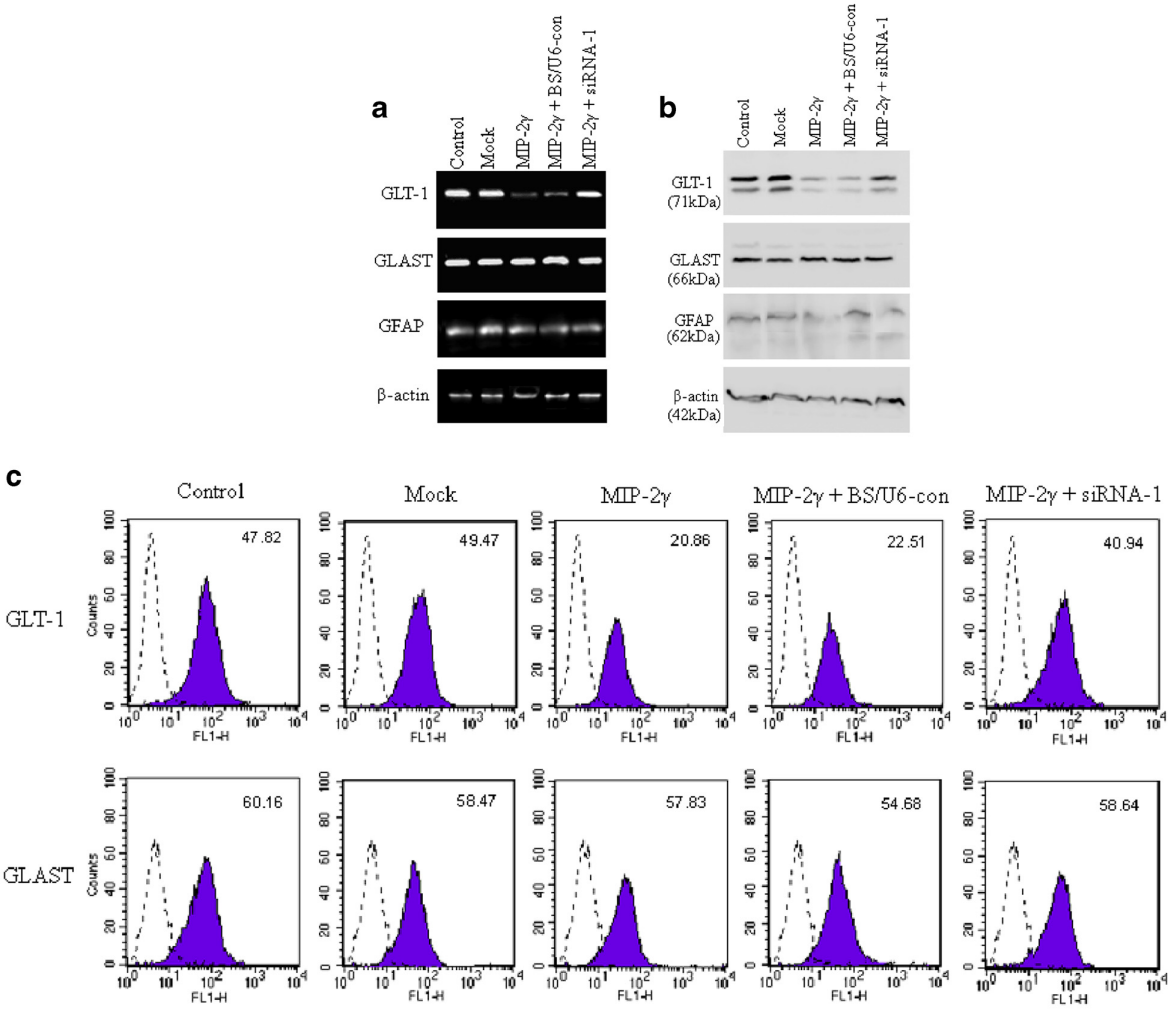
**GLT-1 expression is down-regulated by MIP-2γ in primary cortical astrocytes.** (**a**) Levels of GLT-1 mRNA were decreased in primary astrocytes transfected with pAAV-MIP-2γ-hrGFP plasmids (MIP-2γ) compared with untreated cultures (control) or transfected with pAAV-IRES-hrGFP (mock) vector. MIP-2γ knockdown with siRNA-1 (MIP-2γ + siRNA-1), but not with pBS/U6-con (MIP-2γ + BS/U6-con) inhibits this down-regulation of GLT-1. No significant changes in GLAST, GFAP or β-actin occurred. (**b**) Representative western blot probed for GLT-1, GLAST, GFAP, and β-actin simultaneously. Levels of GLT-1 in astrocytes transfected with pAAV-MIP-2γ-hrGFP plasmids (MIP-2γ) decreased compared with untreated cultures (control) or transfected with pAAV-IRES-hrGFP (mock) vector, which could be partly reversed in astrocytes co-transfected with siRNA-1 (MIP-2γ + siRNA-1) but not with pBS/U6-con (MIP-2γ + BS/U6-con). Levels of GLAST and GFAP were unaffected in all groups. To ensure equal protein loading, blots were reprobed with β-actin-specific antibodies. (**c**) FACS analysis of GLAST or GLT-1 surface expression on astrocytes. Binding of GLAST or GLT-1 (shadow) compared with that of a rat isotype control IgG (dashed line) to intact untreated cultures (control), transfected with pAAV-IRES-hrGFP (mock) vector, transfected with pAAV-MIP-2γ-hrGFP plasmids (MIP-2γ), or pAAV-MIP-2γ-hrGFP plasmids co-transfected with siRNA-1 (MIP-2γ + siRNA-1) or pBS/U6-con (MIP-2γ + BS/U6-con) astrocytes. FACS, fluorescence-activated cell sorting; GFAP, glial fibrillary acidic protein; GLAST, glutamate-aspartate transporter; GLT-1, glutamate transporter; IgG, immunoglobulin G; MIP-2γ, macrophage inflammatory protein-2γ; siRNA, small interfering RNA.

Treatment of astrocytes with dBcAMP increases both GLT-1 and GLAST expression [16]. Our astrocyte cultures are routinely cultured with 250 μM dBcAMP, so GLAST and GLT-1 are both expressed (data not shown). Transfection with pAAV-MIP-2γ-hrGFP reduced GLT-1 expression but not GLAST, and this decrease could be partly reversed by MIP-2γ siRNA-1 (Figure 3a). Flourescence- activated cell sorting analysis showed that transfection of pAAV-MIP-2γ-hrGFP decreased cell-surface GLT-1 expression, which was again partly reversed by MIP-2γ siRNA-1, but did not affect GLAST expression (Figure 3b). Western blots also showed that MIP-2γ overexpression decreased total cellular GLT-1 expression but not GLAST expression (Figure 3c). The actin band provides an index of intracellular proteins present in each preparation, and the cytoskeletal protein, GFAP, confirms the cells are astrocytes.

**Figure 4:**
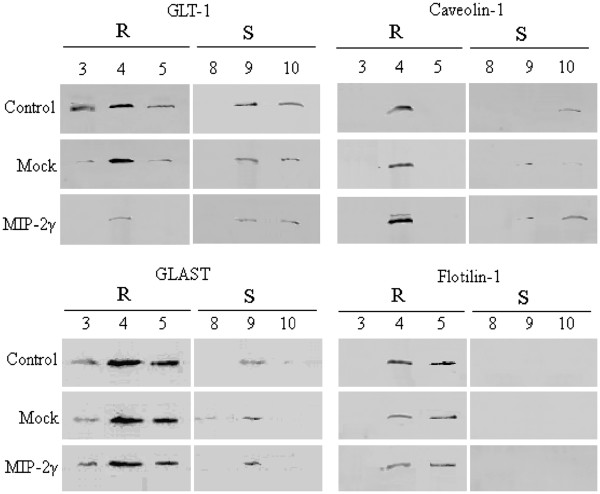
**Overexpression of MIP-2γ inhibits GLT-1 expression in lipid rafts and increases caveolin-1 expression.** Equal amounts of proteins from collected fractions were separated by SDS-PAGE and analyzed by immunoblotting using antibodies against GLT-1, GLAST, flotillin-1, and caveolin-1. Raft domains (R) are recovered in low-density fractions 3, 4 and 5, which are enriched in flotilin-1, a component of lipid rafts. Fractions 8, 9, and 10 correspond to detergent-soluble material (S). Co-fractionation of GLAST and GLT-1 with the lipid raft marker flotillin-1 and caveolin-1 after sucrose density ultracentrifugation. Control, untreated cultures. Mock, astrocytes transfected with pAAV-IRES-hrGFP vector. MIP-2γ, astrocytes transfected with pAAV-MIP-2γ-hrGFP plasmids. GLAST, glutamate-aspartate transporter; GLT-1, glutamate transporter; IgG, immunoglobulin G; MIP-2γ, macrophage inflammatory protein-2γ.

GLT-1 molecules are organized on lipid rafts in astrocytes, which may be necessary for efficient glutamate uptake [18,19]. To isolate these rafts, we performed sucrose gradient ultracentrifugation of detergent-free cell extracts of MIP-2γ-transfected cells in the presence or absence of MIP-2γ siRNA-1 plasmids (48 hours). Immunoblots of pooled fractions corresponding to the raft domains, as confirmed by the presence of the protein, flotillin-1, and detergent-soluble material showed that both GLAST and GLT-1 were recovered in rafts (Figure 4). MIP-2γ overexpression reduced levels of GLT-1 in the raft domains, but did not affect GLAST recruitment into raft domains (Figure 4). Interestingly, MIP-2γ overexpression increased caveolin-1 levels but did not affect flotilin-1 levels (Figure 4), suggesting that the down-regulation of GLT-1 was specific.

### Effects of MIP-2γ on glutamate transport activity

**Figure 5:**
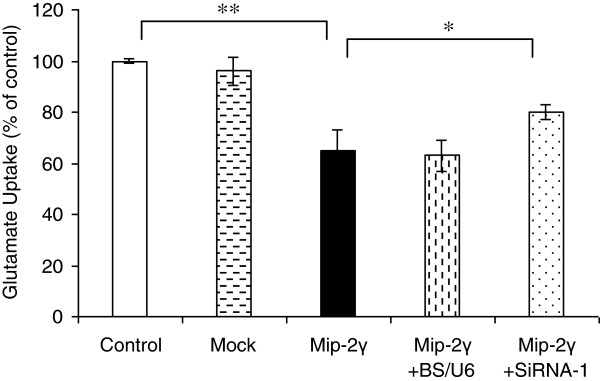
**Effects of MIP-2γ overexpression on astrocyte glutamate uptake.** Results are expressed as the ratio of [^3^H]glutamate uptake of transfected cultures over the value of the control cultures ± SEM. Glutamate uptake capacity was decreased in astrocytes transfected with pAAV-MIP-2γ-hrGFP plasmids (MIP-2γ) compared with untreated cultures (control) or transfected with pAAV-IRES-hrGFP (mock) vector. Astrocytes co-transfected with pAAV-MIP-2γ-hrGFP plasmids and siRNA-1 (MIP-2γ + siRNA-1), but not with pBS/U6-con (MIP-2γ + BS/U6-con), showed partially restored glutamate uptake. n = 3, *P <0.05; **P <0.01 significantly different from the value obtained in control (ANOVA followed by a Bonferroni test). ANOVA, analysis of variance; MIP-2γ, macrophage inflammatory protein-2γ; SEM, standard error of the mean; siRNA, small interfering RNA.

We next measured the uptake of [^3^H]glutamate in astrocytes to determine whether the changes in GLT-1 expression had functional consequences. Na^+^-dependent accumulation of glutamate was measured in untreated astrocytes and in astrocytes overexpressing MIP-2γ in the presence or absence of MIP-2γ siRNA-1 (48 hours). MIP-2γ expression decreased [^3^H] glutamate uptake by about 45% (Figure 5).

### Signaling events linking MIP-2γ overexpression to GLT-1 reduction

**Figure 6:**
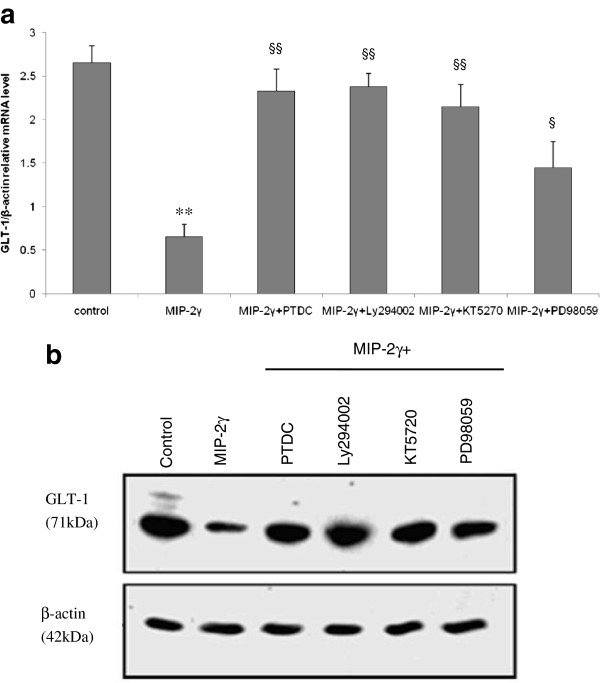
**Effect of pharmacological inhibitors on GLT-1 mRNA and protein expression.** GLT-1 mRNA (**a**) and protein (**b**) expression in astrocytes either untreated (control) or transfected with pAAV-MIP-2γ-hrGFP plasmids (MIP-2γ) in the absence or presence (+) of PDTC (100 μM), LY294002 (10 μM), KT5720 (5 μM), or PD98059 (50 μM) for 48 hours. Individual experiments were performed three times with triplicate samples. Results were expressed as fold of corresponding mean ± SEM after normalization to β-actin. *P <0.05; **P <0.01 compared with control group; ^§^*P* <0.05; ^§§^*P* <0.01 compared with MIP-2γ group; one-way ANOVA, Tukey test. ANOVA, analysis of variance; GLT-1, glutamate transporter; MIP-2γ, macrophage inflammatory protein-2γ; SEM, standard error of the mean.

We next used signaling pathway-specific inhibitors to determine which pathways mediated the decreases in GLT-1 protein levels. MIP-2γ downregulation of GLT-1 expression was eliminated by PDTC, LY294002, KT5720, and partially rescued by PD98059 (Figure 6), suggesting a role for NF-κB, PI-3 K, PKA, and MEK/ERK.

### MIP-2γ influences glutamate neurotoxicity

**Figure 7:**
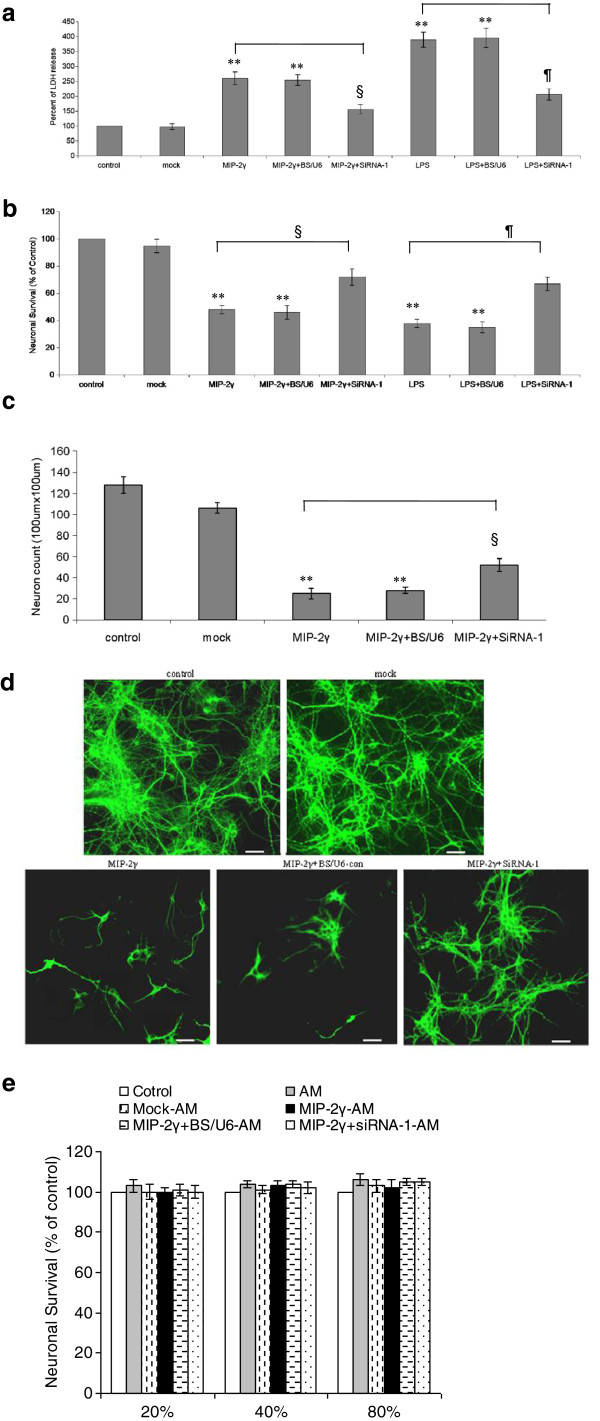
**Over-expression of MIP**-**2γ makes neurons more sensitive to glutamate toxicity.** Cortical astrocytes left untreated (control) or transfected with the plasmid pAAV-IRES-hrGFP (mock) or with pAAV-MIP-2γ-hrGFP (MIP-2γ), or together with siRNA-1 (MIP-2γ +siRNA-1) or with pBS/U6 (MIP-2γ + BS/U6), or treated with LPS (LPS) or together transfected with siRNA-1 (LPS + siRNA-1) or with pBS/U6 (LPS+ BS/U6). Neurons were co-cultured with untreated or treated astrocytes for 4 days; after this time, glutamate (50 μM) was added and, 4 hours later, the neuronal survival was determined using an LDH release assay (**a**) and an MTT assay (**b**) and normalized to normal N/A co-cultures. (**c**,**d**) In parallel experiments, cell viability was assessed by immunocytochemistry using a MAP-2 antibody to detect neurons. (**e**) Astrocyte conditioned media (AM) derived from different groups was applied to neuronal cell cultures and the neuronal survival was determined using an MTT assay. Results are expressed as the percentage of surviving neurons compared with control cultures (control). Mock–AM, AM derived from pAAV-IRES-hrGFP transfected astrocytes. MIP-2γ–AM, AM derived from pAAV-MIP-2γ-hrGFP transfected astrocytes. MIP-2γ + BS/U6-con –AM, AM derived from astrocytes co-transfected pAAV-MIP-2γ-hrGFP with pBS/U6-con. MIP-2γ+siRNA-1 –AM, AM derived from astrocytes co-transfected pAAV-MIP-2γ-hrGFP with siRNA-1. Shown are the mean ± SEM for three experiments. *P <0.05; **P <0.01 compared with the control group; ^§^*P* <0.05 compared with the MIP-2γ group; ^¶^*P* <0.05 compared with the LPS group (ANOVA followed by a Bonferroni test). ANOVA, analysis of variance; LPS, lipopolysaccharide; MAP-2, microtubule-associated protein 2; MIP-2γ, macrophage inflammatory protein-2γ; MTT, 3-(4,5-dimethylthiazol-2-yl)- 2,5-diphenyltetrazolium bromide; SEM, standard error of the mean; siRNA, small interfering RNA.

We used a neuron/astrocytes (N/A) co-culture system to measure glutamate neurotoxicity. Neurons (at 11 days *in vitro*) were co-cultured with differently treated astrocytes for 4 days and then treated with 50 μM glutamate for 4 hours, after which astrocytes were removed and the neuron survival was measured by LDH release and MTT assays. Glutamate was more toxic to neurons in co-culture with MIP-2γ-transfected or LPS-treated astrocytes than normal control astrocytes (Figure 7a, 7b), but MIP-2γ siRNA-1 inhibited this change. Immunocytochemistry using a MAP-2 antibody to detect neurons confirmed these results (Figure 7c, 7d). Thus, MIP-2γ overexpression in astrocytes makes neurons more sensitive to glutamate toxicity by reducing GLT expression and activity. 

In addition, MIP-2γ did not change the effects of astrocyte-conditioned media (AM) on neurons exposed to glutamate toxicity (Figure 7e), suggesting that MIP-2γ alone did not damage neurons nor make neurons more sensitive to glutamate toxicity directly.

## Discussion

The precise physiological function of chemokines in the brain has not yet been fully determined. For example, we have reported that a novel CXC chemokine, MIP-2γ, is widely and constitutively expressed in normal brain [13] and in EAE mice at the onset and relapse phases [14], but little is known about its location or temporal production*.* Here, we investigated the expression of MIP-2γ in astrocytes, a major source of chemokines in the brain, in response to stimulation with LPS or TNF-α, which have been linked to CNS diseases from EAE to AIDS dementia. Optimal MIP-2γ production occurred when confluent astrocyte cultures were stimulated with 5 μg/ml of LPS or 50 ng/ml TNF-α during a 48-hour culture, with peak mRNA occurring after 6 hours of culture (Figure 1b). Thus, MIP-2γ, like other cytokines, is produced by cells intrinsic to the brain: primarily astrocytes, but potentially microglial cells and endothelial cells as well.

Chemokines regulate leukocyte trafficking to the CNS, but are also involved in pathophysiological processes other than chemotaxis [21-23]. Here, we demonstrated for the first time that MIP-2γ overexpression decreases GLT-1 expression (mRNA and protein) and glutamate uptake in primary cortical astrocytes, an effect that could be blocked by MIP-2γ siRNA (Figure 3), but did not affect GLAST expression. MIP-2γ overexpression also reduced the levels of GLT-1 proteins in lipid rafts (Figure 4), which are critical to transporter function [19]. MIP-2γ overexpression actually enhanced caveolin-1 levels. Activation of cAMP-dependent signaling pathways using dBcAMP or application of TGF-α can reduce the expression of caveolin-1 and increase GLT-1 expression in rat astrocytes [19], suggesting a reciprocal regulation of the two proteins in primary astrocytes.

Excessive glutamate stimulation is excitotoxic to neurons, and astrocytes protect against glutamate neurotoxicity by removing extracellular glutamate via glutamate transporter activity. The overexpression of MIP-2γ by astrocytes down-regulated GLT-1 expression and reduced glutamate uptake (Figure 5). We found pharmacological inhibitors, specifically PDTC (an inhibitor of NF-κB activation), LY294002 (a PI-3 K inhibitor), KT5720 (a protein kinase A (PKA) inhibitor), and PD98059 (a MEK/ERK inhibitor) blocked MIP-2γ-mediated changes in GLT-1 expression. Taken together, these results support the hypothesis that MIP-2γ decreases GLT-1 activity in astrocytes through NF-κB, PKA, PI3K, and partly through MEK/ERK signal transduction pathways.

We used two model systems to evaluate whether MIP-2γ overexpression changed glutamate neurotoxicity. The first, a co-culture of astrocytes and neurons, models their interaction to modulate effects of glutamate. Neurons withdrawn from co-culture with MIP-2γ-transduced astrocytes were more sensitive to glutamate toxicity than mock-transduced cells. The second model involves use of conditioned medium from astrocytes, which did not alter neuronal sensitivity to glutamate toxicity. These results suggest that MIP-2γ itself is not toxic, but that the enhanced neuronal sensitivity to excitotoxicity was caused by the decreased GLT-1 activity induced by MIP-2γ.

In conclusion, chemokines and their receptors are primarily involved in regulating CNS inflammatory processes. MIP-2γ may provide a new pathway for neuron-glia communications that are relevant to both normal brain function and neuroinflammatory and neurodegenerative diseases. Furthermore, this research should reveal the potential utility of chemokines and their receptors as targets for therapeutic intervention in CNS disease.

## Abbreviations

ANOVA: analysis of variance; CNS: central nervous system; dBcAMP: dibutyryl cAMP; BSA: bovine serum albumin; DC: dendritic cell; (D)MEM: (Dulbecco’s) modified Eagle’s medium; EAAT: excitatory amino acids transporters; EAE: experimental autoimmune encephalomyelitis; ELISA: enzyme-linked immunosorbent assay; FCS: fetal calf serum; GFAP: glial fibrillary acid protein; GLAST: glutamate-aspartate transporter; GLT: glutamate transporter; IgG: immunoglobulin G; IL-1: interleukin-1; IP-10: inflammatory protein-10; LDH: lactate dehydrogenase; LPS: lipopolysaccharide; MAP2: microtubule-associated protein 2; MIP-2γ: macrophage inflammatory protein-2 gamma; MTT: 3-(4,5-dimethylthiazol-2-yl)- 2,5-diphenyltetrazolium bromide; PBS: phosphate-buffered saline; RT-PCR: reverse transcriptase –polymer chain reaction; SEM: standard error of the mean; siRNA: small interfering RNA; TNF-α: tumor necrosis factor-α.

## Competing interests

The authors declare they have no competing financial interests.

## Authors’ contributions

DH, JF, JH and QT carried out the cell culture, laboratory work, and analyses; DH and YT contributed to the design of the study; YT and DH provided the research support. JF, JH, and DH wrote the manuscript. All authors read and approved the final version of the manuscript.

## References

[B1] Beart PM O’Shea RD Transporters for l-glutamate: an update on their molecular pharmacology and pathological involvement Br J Pharmacol 2007 150 5 17 17088867 10.1038/sj.bjp.0706949 PMC2013845

[B2] Kaul M Garden GA Lipton SA Pathways to neuronal injury and apoptosis in HIV-associated dementia Nature 2001 410 988 994 10.1038/35073667 11309629

[B3] Mitosek-Szewczyk K Sulkowski G Stelmasiak Z Struzyńska L Expression of glutamate transporters GLT-1 and GLAST in different regions of rat brain during the course of experimental autoimmune encephalomyelitis Neuroscience 2008 155 45 52 10.1016/j.neuroscience.2008.05.025 18572325

[B4] Rossi DJ Brady JD Mohr C Astrocyte metabolism and signaling during brain ischemia Nat Neurosci 2007 10 1377 1386 10.1038/nn2004 17965658 PMC8906499

[B5] Shigeri Y Seal RP Shimamoto K Molecular pharmacology of glutamate transporters, EAATs and VGLUTs Brain Res Brain Res Rev 2004 45 250 265 15210307 10.1016/j.brainresrev.2004.04.004

[B6] Halassa MM Haydon PG Integrated brain circuits: astrocytic networks modulate neuronal activity and behavior Annu Rev Physiol 2010 72 335 355 10.1146/annurev-physiol-021909-135843 20148679 PMC3117429

[B7] van Neerven S Nemes A Imholz P Regen T Denecke B Johann S Beyer C Hanisch UK Mey J Inflammatory chemokine release of astrocytes in vitro is reduced by all-trans retinoic acid J Neurochem 2010 114 1511 1526 20557428 10.1111/j.1471-4159.2010.06867.x

[B8] Carpentier PA Begolka WS Olson JK Elhofy A Karpus WJ Miller SD Differential activation of astrocytes by innate and adaptive immune stimuli Glia 2004 49 360 374 15538753 10.1002/glia.20117

[B9] McKimmie CS Graham GJ Astrocytes modulate the chemokine network in a pathogen-specific manner Biochem Biophys Res Commun 2010 394 1006 1011 10.1016/j.bbrc.2010.03.111 20331977

[B10] Dhillon NK Williams R Callen S Zien C Narayan O Buch S Roles of MCP-1 in development of HIV-dementia Front Biosci 2008 13 3913 3918 18508485 10.2741/2979 PMC2715276

[B11] Fuhrmann M Bittner T Jung CK Burgold S Page RM Mitteregger G Haass C LaFerla FM Kretzschmar H Herms J Microglial Cx3cr1 knockout prevents neuron loss in a mouse model of Alzheimer’s disease Nat Neurosci 2010 13 411 413 10.1038/nn.2511 20305648 PMC4072212

[B12] Hamann I Zipp F Infante-Duarte C Therapeutic targeting of chemokine signaling in Multiple Sclerosis J Neurol Sci 2008 274 31 38 10.1016/j.jns.2008.07.005 18706659

[B13] Cao X Zhang W Wan T He L Chen T Yuan Z Ma S Yu Y Chen G Molecular cloning and characterization of a novel CXC chemokine macrophage inflammatory protein-2 gamma chemoattractant for human neutrophils and dendritic cells J Immunol 2000 165 2588 2595 10946286 10.4049/jimmunol.165.5.2588

[B14] Han D Tian Y Zhang M Zhou Z Lu J Prevention and treatment of experimental autoimmune encephalomyelitis with recombinant adeno-associated virus-mediated alpha-melanocyte-stimulating hormone-transduced PLP139-151-specific T cells Gene Ther 2007 14 383 395 10.1038/sj.gt.3302862 17066098

[B15] Sui G Soohoo C Affar el B Gay F Shi Y Forrester WC Shi Y A DNA vector-based RNAi technology to suppress gene expression in mammalian cells Proc Natl Acad Sci U S A 2002 16 5515 5520 11960009 10.1073/pnas.082117599 PMC122801

[B16] Poulsen CF Schousboe I Sarup A White HS Schousboe A Effect of topiramate and dBcAMP on expression of the glutamate transporters GLAST and GLT-1 in astrocytes cultured separately, or together with neurons Neurochem Int 2006 48 657 661 10.1016/j.neuint.2006.01.006 16524645

[B17] Skytt DM Madsen KK Pajęcka K Schousboe A Waagepetersen HS Characterization of primary and secondary cultures of astrocytes prepared from mouse cerebral cortex Neurochem Res 2010 35 2043 2052 10.1007/s11064-010-0329-6 21127969

[B18] González MI Krizman-Genda E Robinson MB Caveolin-1 regulates the delivery and endocytosis of the glutamate transporter, excitatory amino acid carrier 1 J Biol Chem 2007 282 29855 29865 10.1074/jbc.M704738200 17715130

[B19] Zschocke J Bayatti N Behl C Caveolin and GLT-1 gene expression is reciprocally regulated in primary astrocytes: association of GLT-1 with non-caveolar lipid rafts Glia 2005 49 275 287 10.1002/glia.20116 15494979

[B20] Lee ES Sidoryk M Jiang H Yin Z Aschner M Estrogen and tamoxifen reverse manganese-induced glutamate transporter impairment in astrocytes J Neurochem 2009 110 530 544 10.1111/j.1471-4159.2009.06105.x 19453300 PMC3920654

[B21] Florio T Schettini G Chemokines, their receptors and significance in brain function Immune Biol 2008 6 242 273

[B22] Rostène W Guyon A Kular L Godefroy D Barbieri F Bajetto A Banisadr G Callewaere C Conductier G Rovère C Mélik-Parsadaniantz S Florio T Chemokines and chemokine receptors: New actors in neuroendocrine regulations Front Neuroendocrinol 2011 32 10 24 10.1016/j.yfrne.2010.07.001 20624414

[B23] Sanchez A Tripathy D Grammas P RANTES release contributes to the protective action of PACAP38 against sodium nitroprusside in cortical neurons Neuropeptides 2009 43 315 320 10.1016/j.npep.2009.05.002 19497618 PMC2726654

